# The traditional Chinese medicine bloodletting therapy at the Taiyang acupoint (No.EX-HN5) for the treatment of periorbital venous malformation: a case report

**DOI:** 10.3389/fopht.2026.1787288

**Published:** 2026-06-17

**Authors:** Yanli Zhang, Xiaoyun Cao, Chunhong Chen, Zengfang Yu, Dan Li, Chaoqun Zhang, Man Kang

**Affiliations:** Ningbo Key Laboratory of Medical Research on Blinding Eye Diseases, Ningbo Eye Institute, Ningbo Eye Hospital, Wenzhou Medical University, Ningbo, China

**Keywords:** bloodletting therapy, Chinese medicine appropriate technology, meridian stabbing, periorbital, venous malformation

## Abstract

**Background:**

This paper presents a case of periorbital venous malformation treated using appropriate Traditional Chinese Medicine interventions.

**Case presentation:**

A 27-year-old Asian Han female presented with prominent swelling of the right periorbital region that appeared when looking downward, accompanied by greenish skin discoloration, which resolved upon elevating the head. Initially, the patient did not seek medical attention, attributing the symptoms to fatigue and insufficient rest. However, the swelling recurred upon awakening, prompting the patient to seek medical evaluation at a hospital. Ocular ultrasound and orbital computed tomography (CT) were subsequently performed, resulting in a diagnosis of periorbital VM. Ocular ultrasound revealed the VM measured 2.0×1.28 cm. Additional ophthalmological findings were as follows: intraocular pressure-OD 14 mmHg, OS 16 mmHg; refraction-OD 0.00/-0.25, OS -1.00/-0.25; visual acuity-OD 1.0, OS 0.8. The patient was subsequently referred to the Departments of Traditional Chinese Medicine (TCM) and Ophthalmology, where management was overseen by a TCM ophthalmologist. The physician recommended bloodletting at the ear tips or Taiyang acupoints as part of the treatment and instructed the patient to adhere to this regimen for monitoring and further management. The patient complied with the physician’s instructions, undergoing Taiyang acupoint bloodletting once weekly, with approximately 2 mL of blood removed per session. Six months later, follow-up ocular ultrasound demonstrated a reduction in VM size, although minor changes could be attributable to measurement variability. No evidence of lesion growth was observed, prompting further follow-up evaluations. The patient was instructed to abstain from meridian-based Taiyang acupoint bloodletting for two weeks. Serial ocular ultrasound examinations were performed to assess VM size and peripheral blood flow at multiple time points: pre-bloodletting, immediately post-procedure, and at 1, 2, 5, 7, 24 hours, and 3, 7, 12, and 14 days following the intervention. The results indicated the clinical efficacy of combined Chinese and Western interventions, including Taiyang acupoint meridian stabbing and bloodletting therapy, in managing periorbital VM.

**Conclusion:**

Taiyang acupoint meridian bloodletting therapy, when applied to a localized periorbital VM, may exert a therapeutic effect. The therapy temporarily reduces blood flow to the VM, alleviates swelling, improves patient comfort, and mitigates tissue compression associated with the lesion. However, this case report focuses exclusively on a localized periorbital VM during follow-up assessment and is intended solely for clinical reference. Medical guidance should be followed for the targeted and comprehensive management of VM.

## Introduction

Orbital vascular tumors arise from the proliferation of newly formed blood vessels, vascular wall components, and cellular elements associated with vascular tissue origin (1). Common orbital vascular lesions include cavernous VM, capillary VM, and lymphangiomas (2). The etiology of these tumors is likely multifactorial, encompassing genetic factors (3), acute triggers, and other mechanisms (4). Although relatively rare, these diseases can substantially impact patients’ quality of life, often inducing anxiety (5). Periorbital VM may lead to proptosis (6), visual disturbances (7), eyelid edema, and, in severe cases, impairment of normal ocular function (8). The dense and intricate vascular network of the periorbital region can contribute to postoperative complications, such as hemorrhage, infection, scarring, and recurrence (9, 10). Consequently, the safe and effective management of periorbital VM remains a significant clinical challenge.

Recent advances have been achieved in the management of periorbital VM; nevertheless, substantial challenges persist. Currently, the incidence of VM is higher in children than in adults (11), and conventional treatment modalities primarily include pharmacotherapy (12), laser therapy (13), and surgical intervention (14). Several studies have suggested that oral propranolol constitutes the first-line therapy for periorbital VM in pediatric patients, owing to its favorable safety profile (15). Radiosurgical intervention for orbital cavernous VM may reduce the risk of complications (16). In contrast, asymptomatic adult patients with VM are frequently recommended to pursue conservative management (17, 18).

Research investigating the potential role of TCM in the management of periorbital VM is ongoing. Several studies have reported that TCM may alleviate symptoms and reduce recurrence (19). By assessing clinical manifestations and implementing individualized treatment plans, TCM can tailor interventions to the patient’s specific needs, thereby enhancing therapeutic efficacy (20). In China, numerous studies have investigated the application of traditional therapies, including TCM, in ophthalmic diseases, demonstrating notable clinical outcomes (21, 22). For example, TCM formulations designed to promote blood circulation and resolve blood stasis may help alleviate VM-associated symptoms (23). However, Taiyang acupoint-based bloodletting, a distinctive TCM therapeutic approach, has been rarely applied in the management of periorbital VM. Its application is seldom addressed in the current literature. Bloodletting therapy, a well-established technique, is employed to dispel blood stasis and stimulate blood regeneration by puncturing superficial vessels. It is primarily indicated for conditions associated with stagnation, heat, and toxicity (24). According to the TCM principle “the acupoint corresponds to the site of the disease”, the Taiyang acupoint (EX-HN5) is considered particularly effective for treating head and facial disorders (25). Taiyang acupoint bloodletting is performed by puncturing the area between the superior border of the eyebrow and the upper margin of the ear. This technique enhances local circulation, resolves blood stasis at the VM site, and reduces associated swelling and pain. Although this therapy has shown efficacy in managing conditions such as headache (26), blepharitis (27), ocular redness, edema, and pain (28), wheals (29), and acute conjunctivitis (30), its application in ocular VM remains uncommon. Periorbital VM do not have a direct analogue in TCM theory but may be classified as “VM”, “VMs”, “red silk and blood strands”, or “blood stasis” according to clinical presentation. In TCM, the development of these VM is attributed to imbalances in qi and blood, blood heat, phlegm accumulation, and blood stasis, among other factors.

Although Taiyang acupoint-based bloodletting is widely used in TCM, its application in the management of periorbital VM remains under-investigated. This case report aims to evaluate the therapeutic effect of Taiyang acupoint bloodletting in the management of periorbital VM. Taiyang acupoint-based bloodletting may mitigate swelling and associated symptoms of VM by enhancing periorbital circulation and resolving local blood stasis. The objective of this case study is to determine whether Taiyang acupoint-based bloodletting can serve as an adjunctive therapy for periorbital VM through clinical observation and efficacy assessment. Additionally, this study seeks to further validate the feasibility and therapeutic efficacy of this TCM approach within contemporary clinical practice.

## Case presentation

A 27-year-old Asian Han woman noticed a protrusion in the right orbit while tying her shoes at home, which returned to its original shape once she stood up. She sought medical attention at our hospital in December 2024 after observing recurrent swelling in the right orbital region each morning upon waking for one week. Periorbital VM was diagnosed based on ocular ultrasound and CT examination findings. The results of the initial examination are presented in **Table 1**. Ocular ultrasound revealed a VM measuring 2.00×1.28 cm, with a rich blood supply. The CT scan indicated that the VM measured 1.4×1.3×0.8 cm, with intraocular pressures of OD 14 mmHg and OS 16 mmHg. Optometry results showed OD -0.00/-0.25 and OS -1.00/0.00, with visual acuity of OD 1.0 and OS 0.8. Since the VM was relatively small and not visibly prominent, the physician recommended conservative treatment with follow-up examinations every six months.

**Table 1:** Diagnostic test report.

Ultrasound examination of the eye	CT scanning examination
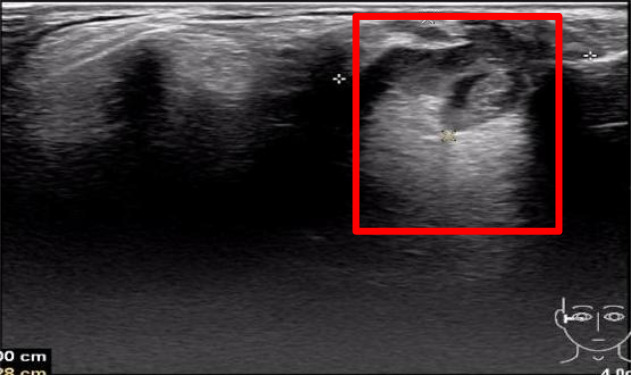 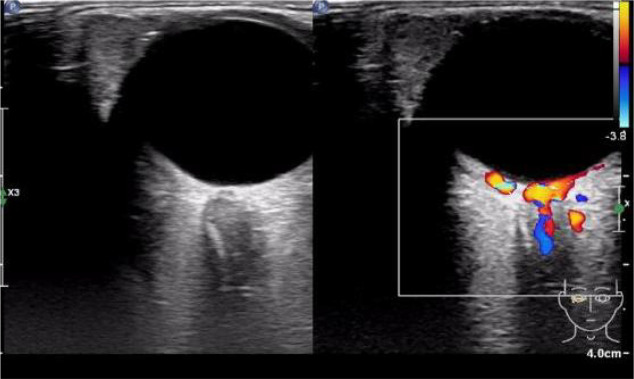	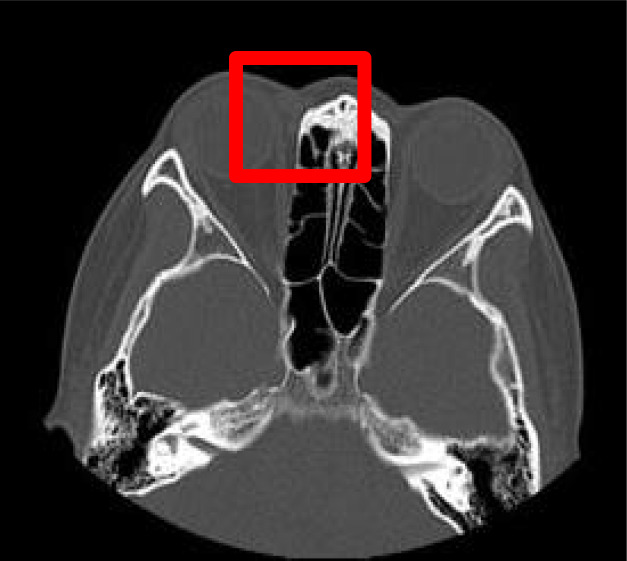 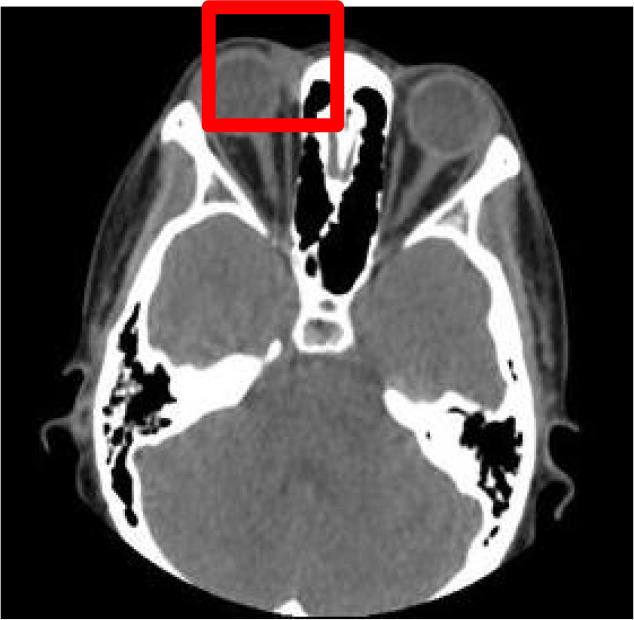 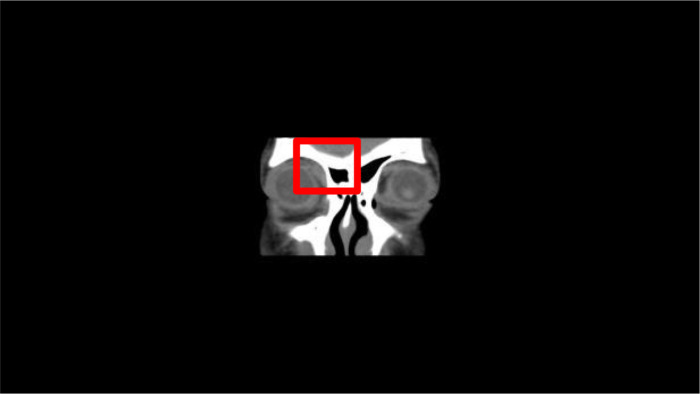
Diagnosis of periorbital mass requiring further examination	The examination revealed a patchy high-density shadow in the soft tissue of the right eye’s inner can thus, with well-defined boundaries and no notable abnormalities in the adjacent bone. The morphology of both eyeballs appeared normal, with intact eye rings, uniform density within the eyeballs, and no significant thickening of the optic nerves bilaterally. The extraocular muscles showed uniform density with no obvious swelling, and the surrounding fat spaces were clear. Additionally, there was no apparent destruction of the orbital wall bone, nor any enlargement of the optic nerve foramina bilaterally. Based on these findings, the physician diagnosed periorbital VM.

The patient subsequently consulted with an ophthalmologist specializing in TCM, who based on clinical experience, recommended treatment options including traditional Chinese medicine fumigation, bloodletting at the ear-tip points, or bloodletting at the Taiyang acupoint points. The patient ultimately chose to proceed with bloodletting therapy at the Taiyang acupoint. The primary reasons for selecting acupuncture bloodletting therapy at the Taiyang acupoint for this patient are as follows: (1) Medications such as propranolol are primarily used for pediatric patients. As the patient is an adult, clinical drug therapy has proven ineffective for this individual (31); (2) The VM is located in a relatively specific position-above the right orbital rim-where it does not compress nerves nor exert pressure or obstruction on any part of the eyeball. The VM is also small in size (32); (3) The patient expressed concerns about discomfort or complications from invasive treatments like laser surgery, opting for conservative management within the limited scope of the small VM (33); (4) After reviewing literature and texts, the TCM ophthalmologist determined that VM in this specific location could be treated via blood-letting at the Taiyang acupoint along the blood meridian. Bloodletting therapy, a traditional treatment in Traditional Chinese Medicine, has demonstrated preliminary efficacy in treating various vascular and neoplastic diseases in recent years (34, 35). In the treatment of periorbital VM, clinical experience suggests that bloodletting therapy can alleviate symptoms by reducing local blood stasis (36), promoting the circulation of qi and blood (37), and is particularly effective for patients in the early stages or those with mild symptoms (38). Bloodletting therapy is performed by selecting specific local points, which directly enhances blood circulation, reduces local swelling and compression, and alleviates pain and discomfort associated with vasodilation (39).

Six months after the treatment, ultrasound examination revealed no increase in the size of the periorbital VM. The ultrasound results revealed the VM’s dimensions as 1.70×1.67×1.27 cm, with intraocular pressures of OD 14 mmHg and OS 16 mmHg. The optometry results showed OD -0.00/-0.25 and OS -1.00/0.00, with visual acuity of OD 1.0 and OS 0.8. Given the unique location of the periorbital VM, which is suitable for bloodletting therapy via meridian stabbing at the Taiyang acupoint, the size of the VM and changes in blood flow were closely monitored. Prior to monitoring, the patient’s blood cells were examined, and no contraindications for bloodletting therapy, such as anemia, were found. The patient’s pulse was also evaluated, and her overall constitution was determined to be suitable for Taiyang acupoint bloodletting therapy (40). **Table 2** presents the patient’s blood test report.

**Table 2:** Complete blood count report.

Blood tests		
White blood cell count	5.2×10^9^/L	Reference value 3.5-9.5
Erythrocyte count	4.62×10^12^/L	Reference value 3.8-5.1
Hemoglobin	142 g/L	Reference value 115-150
Erythrocyte Compaction	41.9% of the total	Reference value 35-45
Platelet count	264×10^9^/L	Reference value 125-350
Platelet Compaction	0.25% of the total platelet count	Reference value 0.11-0.28
Mean red blood cell volume	90.9 fL	Reference value 82-100
Mean RBC hemoglobin volume	30.8 pg	Reference value 27-34
Mean RBC hemoglobin concentration	339g/L	Reference value 316-354
Red cell distribution width (RD) W-CV)	12.2% RD	Reference value 11-14.5
Mean platelet volume	9.3 fL	Reference value 6.5-13
Platelet distribution width	16.0%	Reference value 10-16

According to literature and guidelines, the volume of blood drawn should be determined based on individual conditions, typically ranging from 2 to 7 ml. Treatment frequency and cupping retention time should also be established (41–44). The specific clinical procedure for this case was as follows: The patient assumed a sitting position. The physician selected the Taiyang acupoint on the right sample, disinfected the surrounding skin, and used a needle to perform a shallow puncture at the acupoint until minimal bleeding occurred. After bleeding, a cup was applied and retained for 5 minutes. The treatment for this patient was administered biweekly, with each session involving approximately 2 mL of bloodletting. After bloodletting, the skin around the acupoint was disinfected again. The patient was informed that bruising at the site was a normal occurrence. Based on the patient’s condition, we developed a comprehensive examination plan to assess the size of the periorbital VM via orbital ultrasound and monitor blood flow changes at various time points: prior to bloodletting, immediately after, 1 hour, 2 hours, 5 hours, 7 hours, 24 hours, 3 days, 7 days, 12 days, and 14 days post-bloodletting. The specific bloodletting tools used, as well as changes in the patient’s orbital appearance and Taiyang acupoint points after bloodletting, are shown in **Table 3**.

**Table 3:** Tools and patient pictures.

Bloodletting tools	Orbital area before bloodletting	Orbital area after bloodletting	Taiyang acupoint points
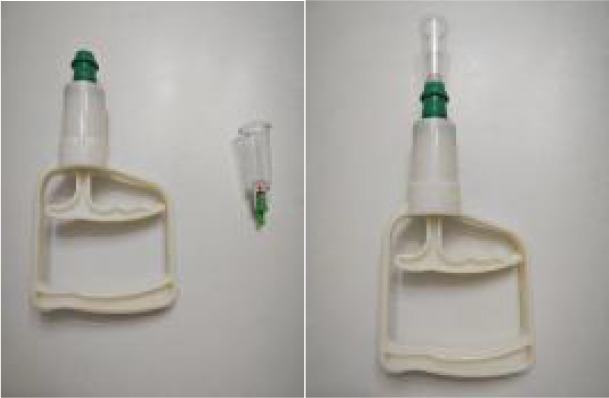	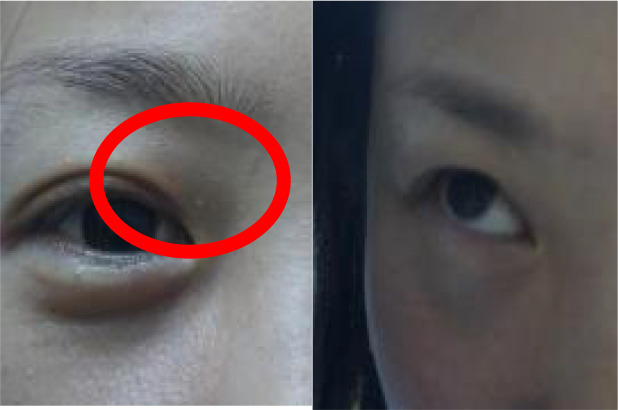	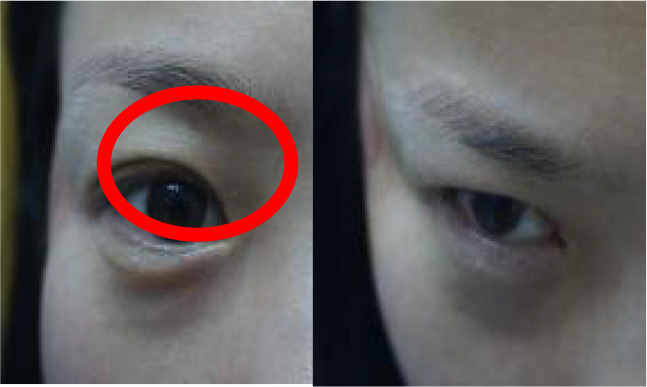	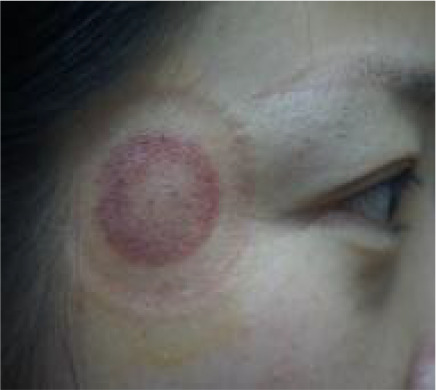

**Figure 1:**
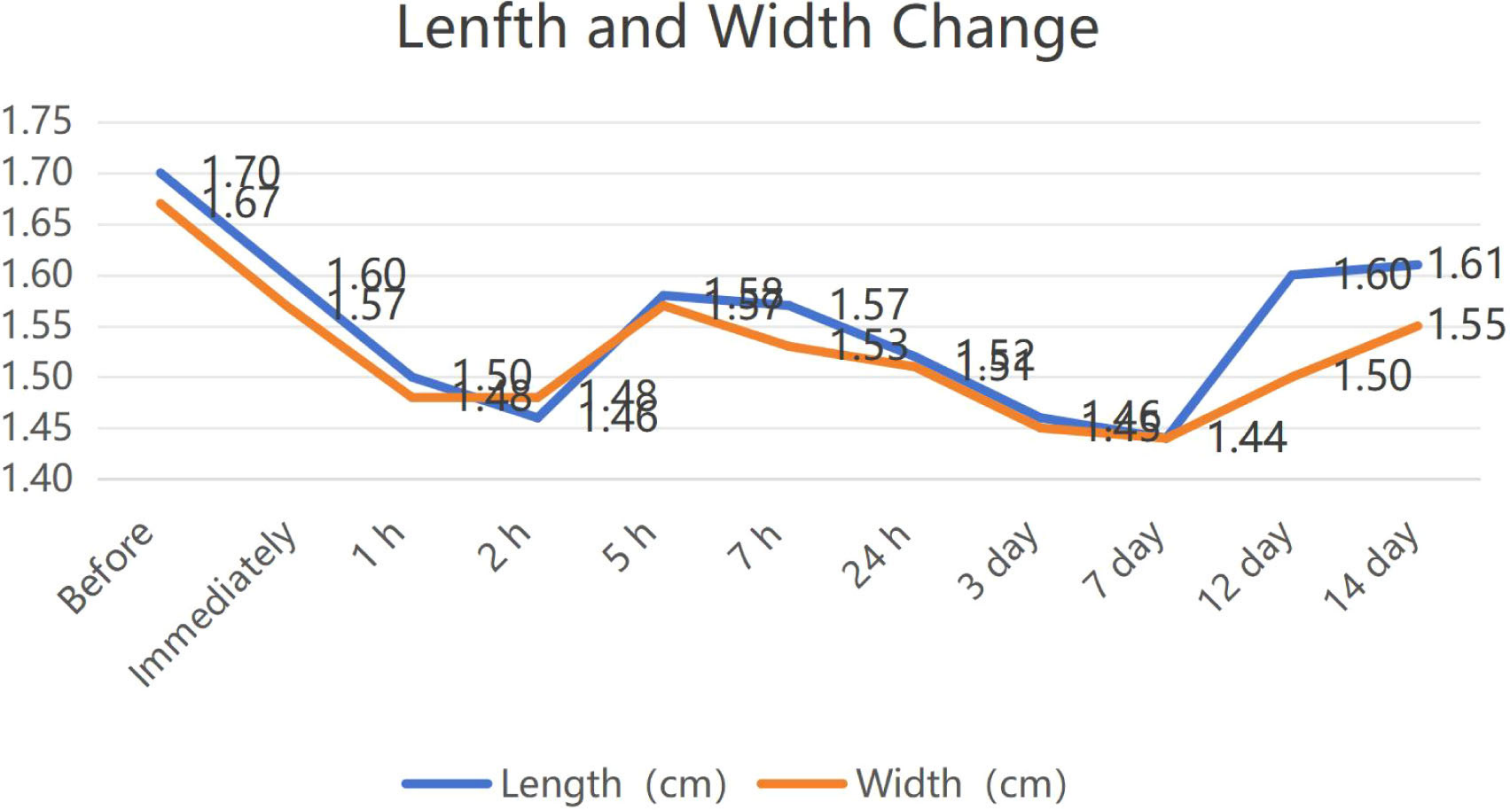
VM length and width change.

**Figure 2:**
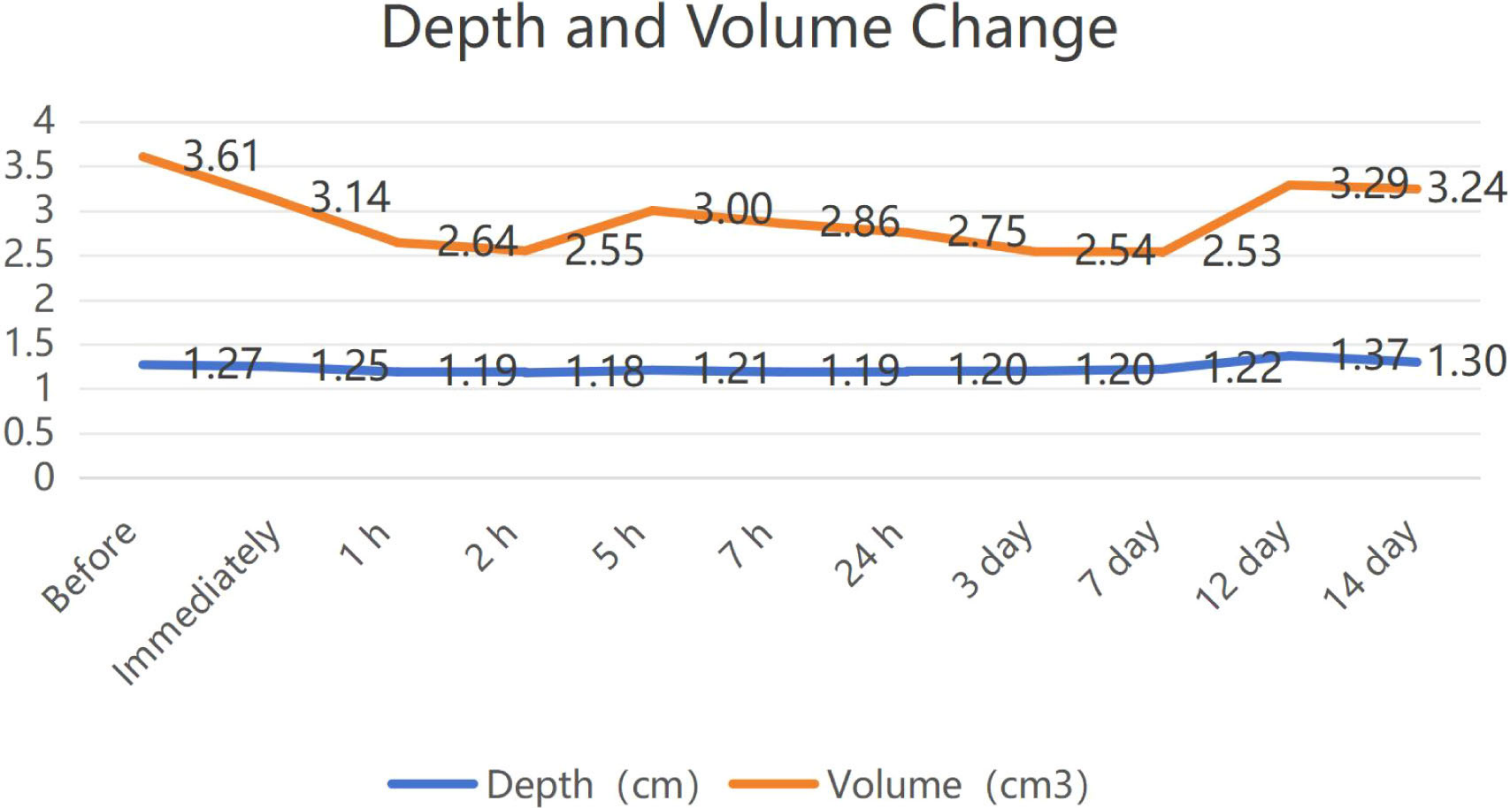
VM depth and volume change.

To ensure the accuracy of the results, all measurements during this inspection were performed by the same inspector using the same testing instrument. The changes in the periorbital VM before and after bloodletting are presented in **Table 4**. The variations in the size of the VM are depicted in **Figures 1**, **2**.

**Table 4:** Ultrasound examination report.

Time	Size of color ultrasound	Ultrasound blood supply
Before bloodletting	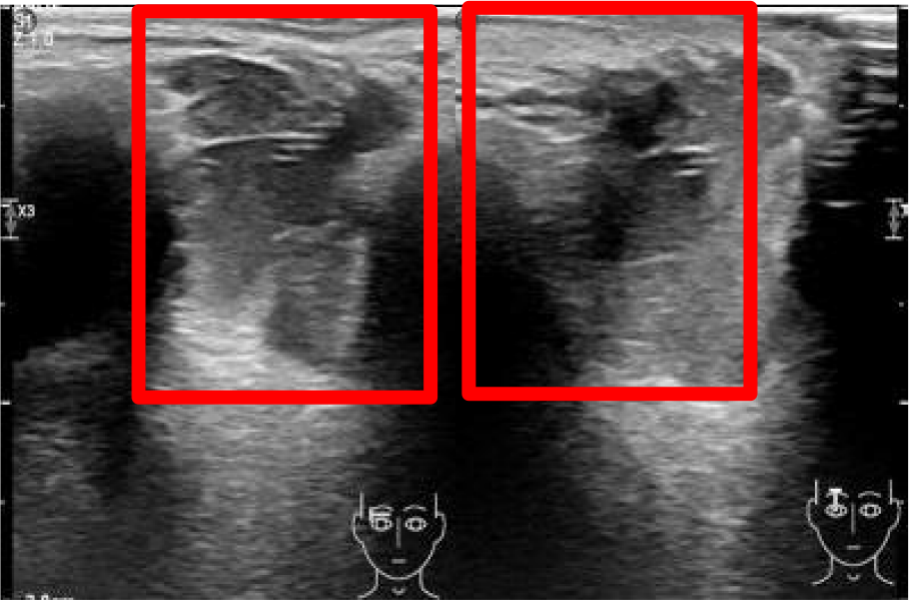	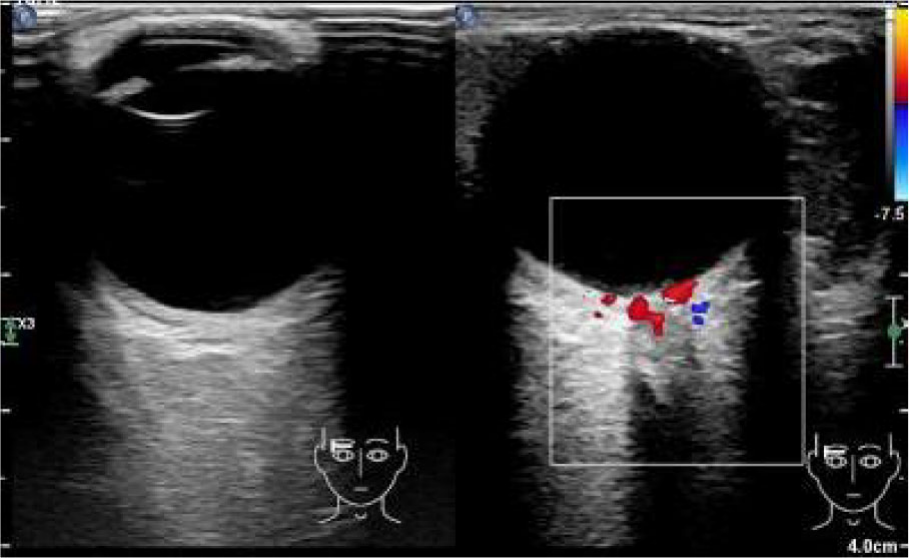
Immediately after bloodletting	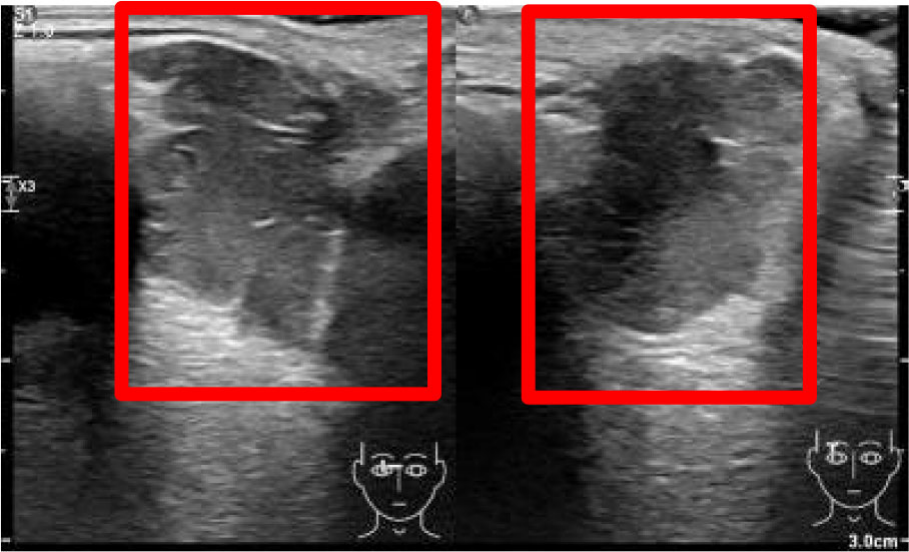	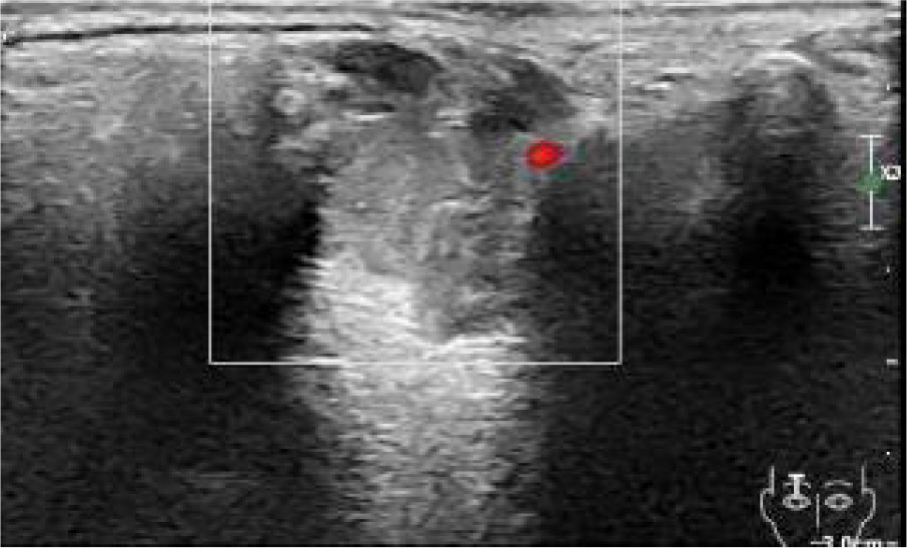
1h	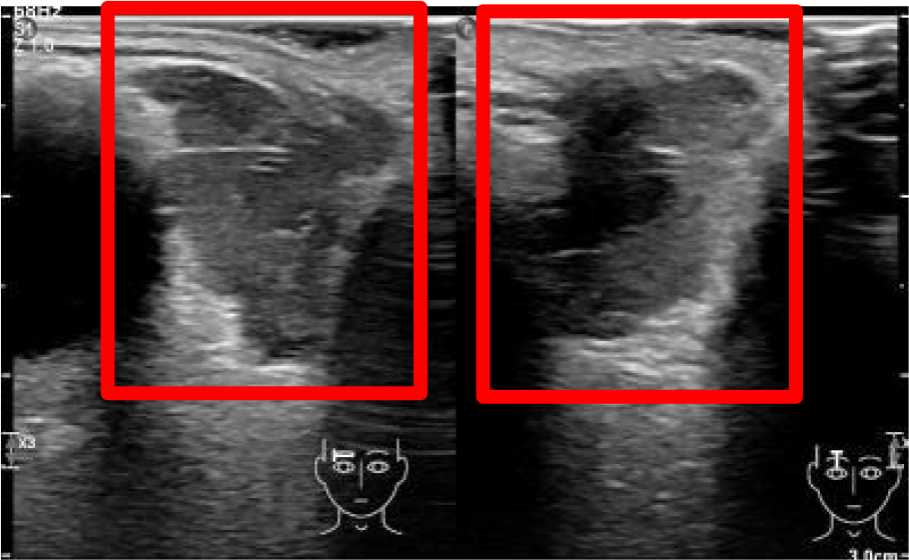	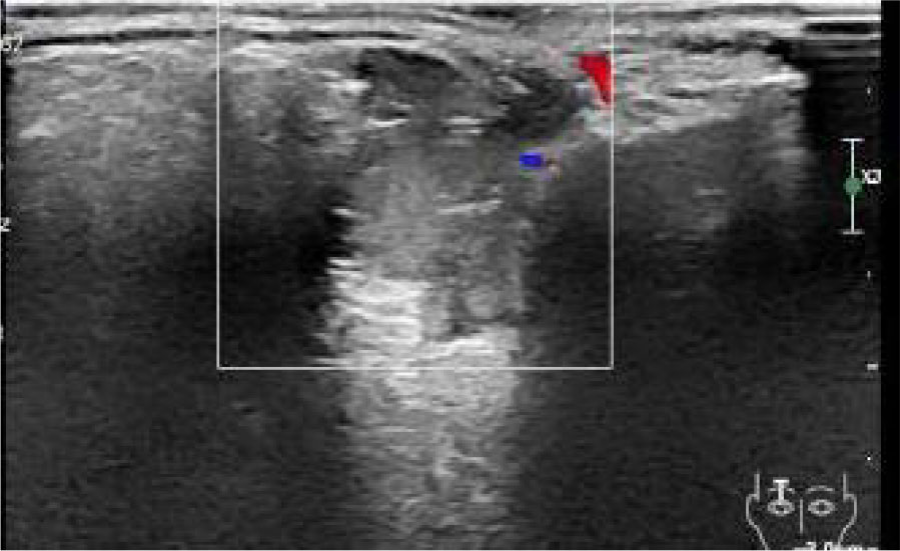
2h	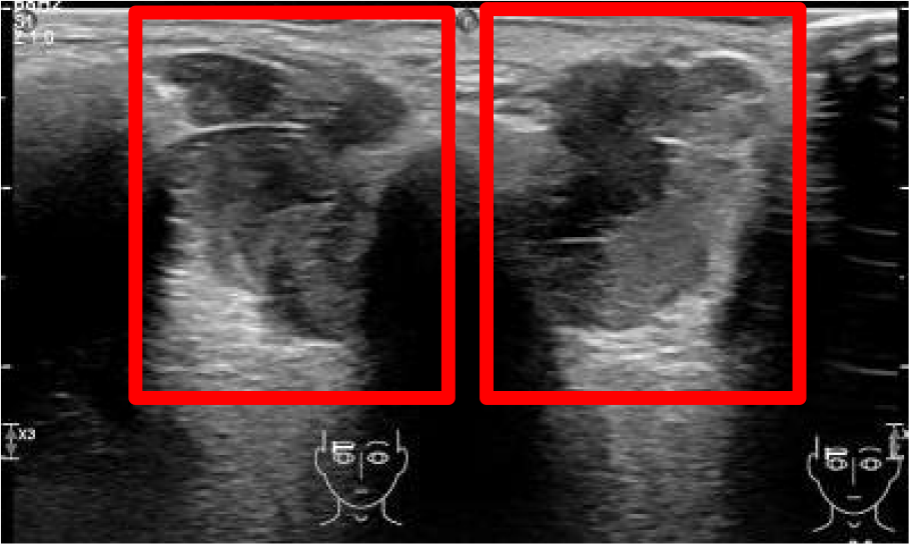	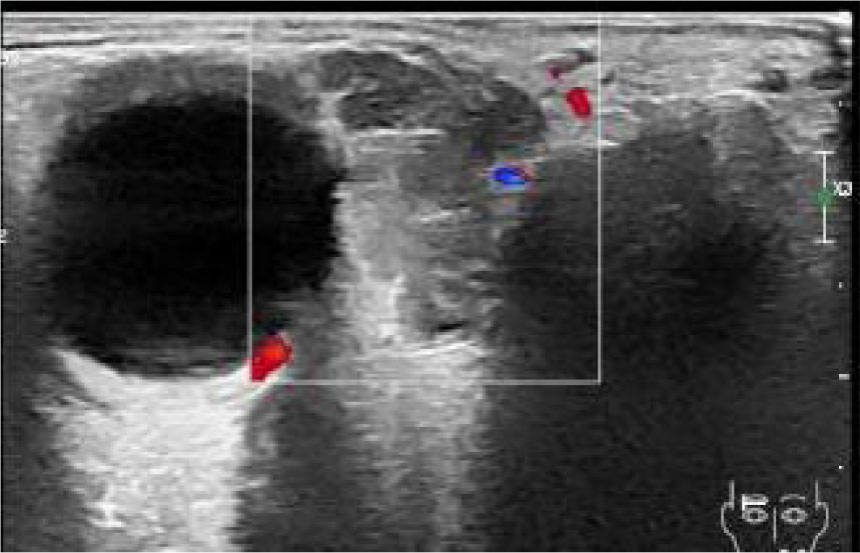
5h	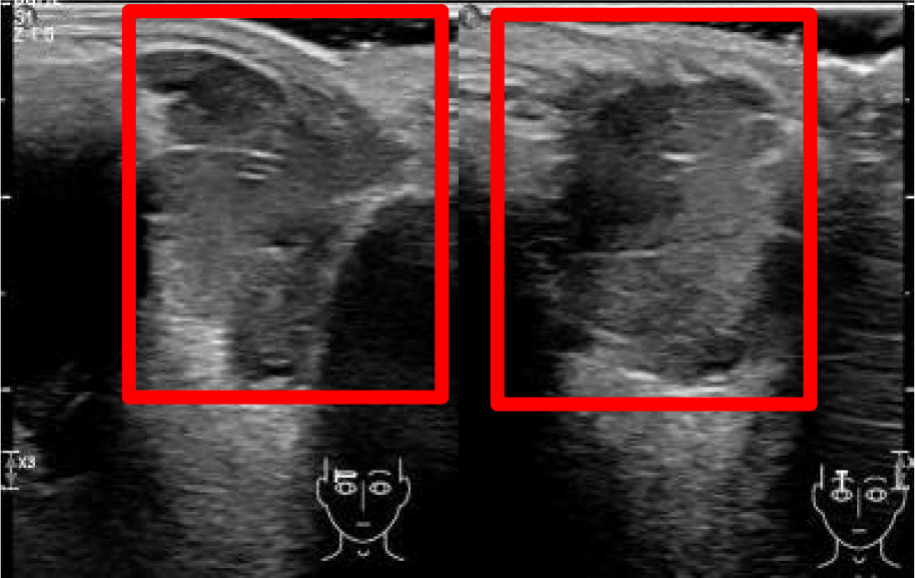	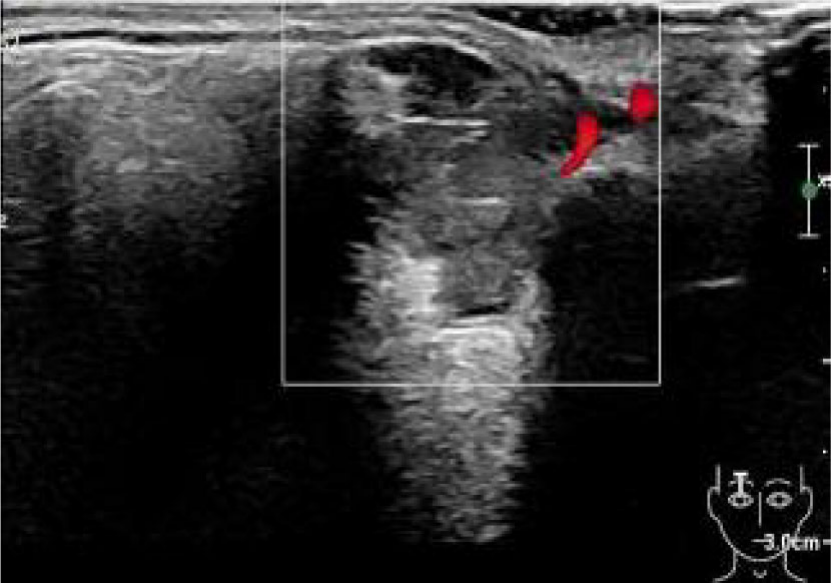
7h	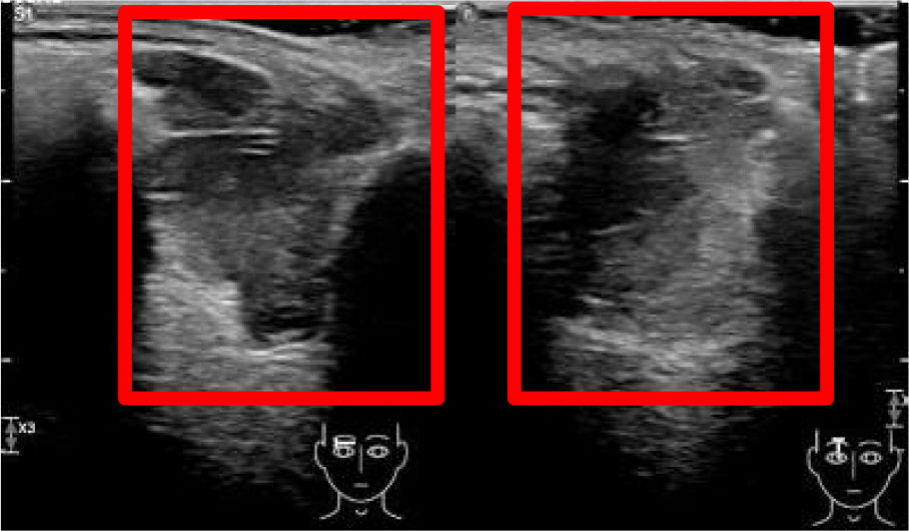	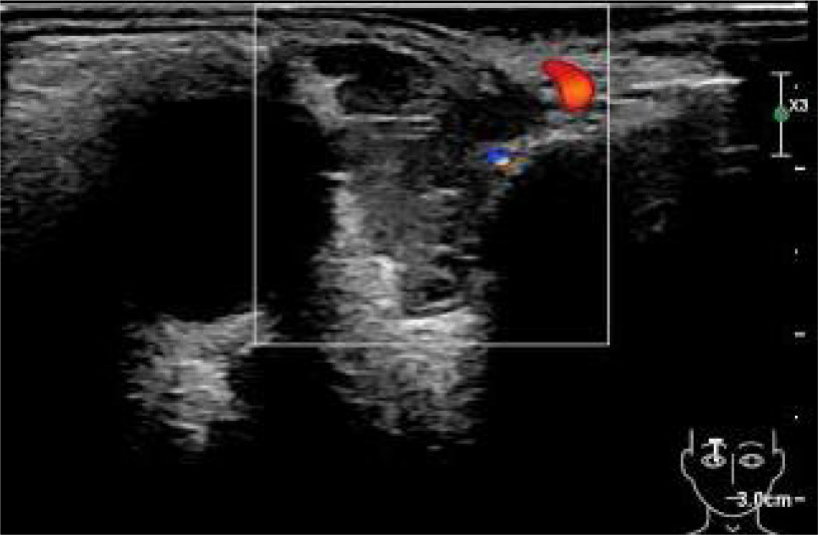
24h	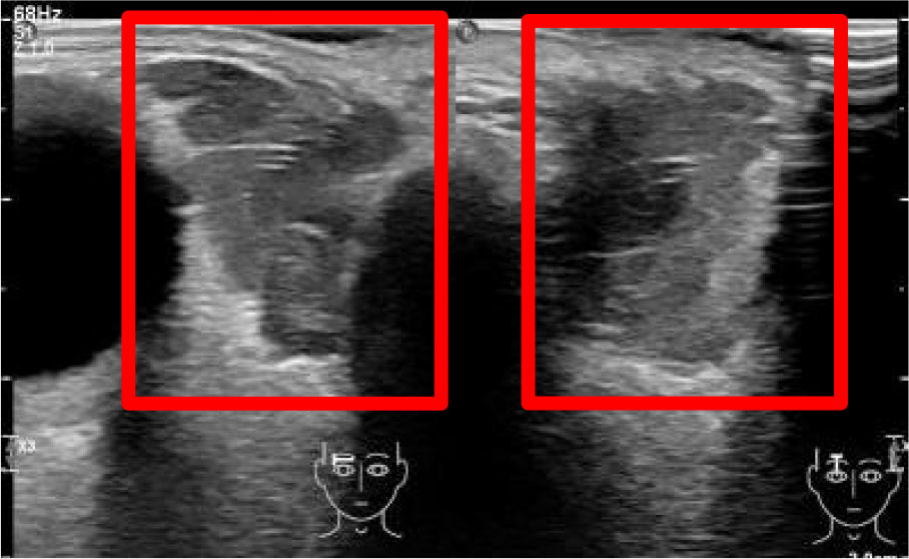	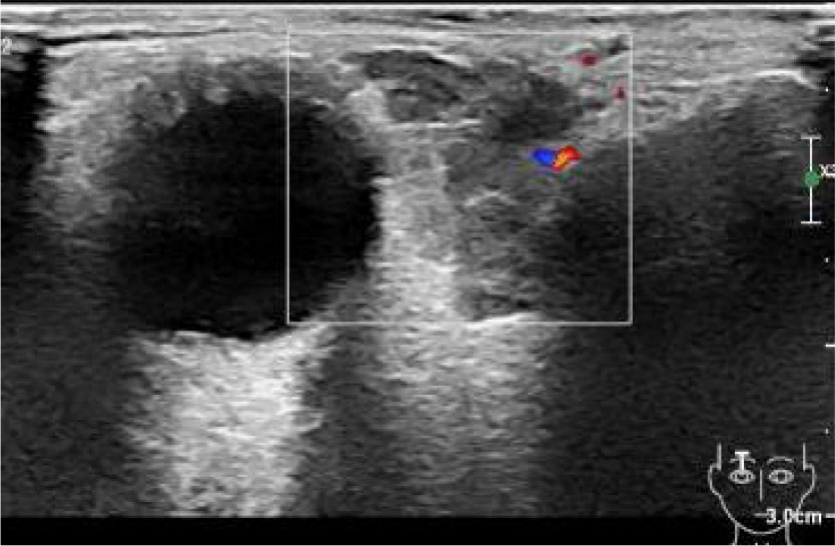
3 days	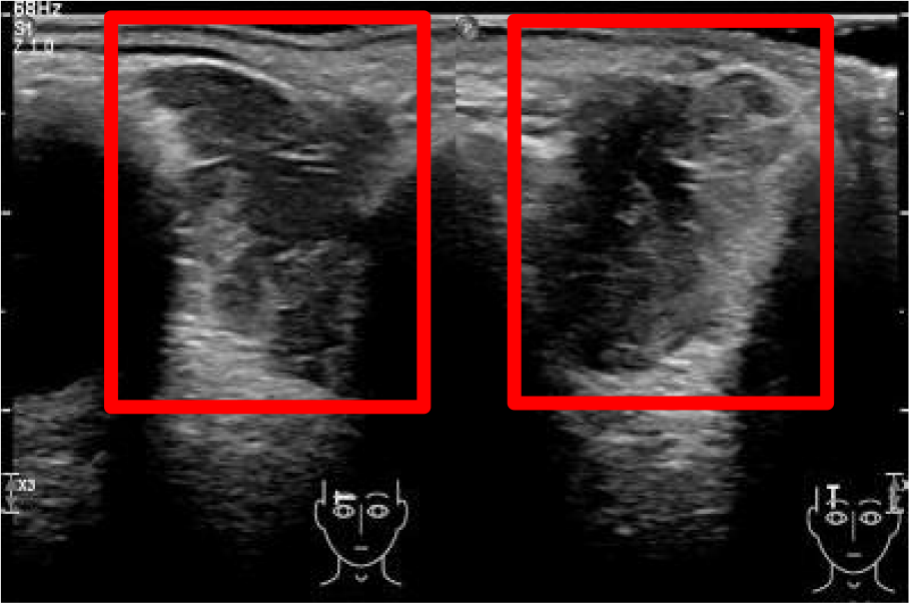	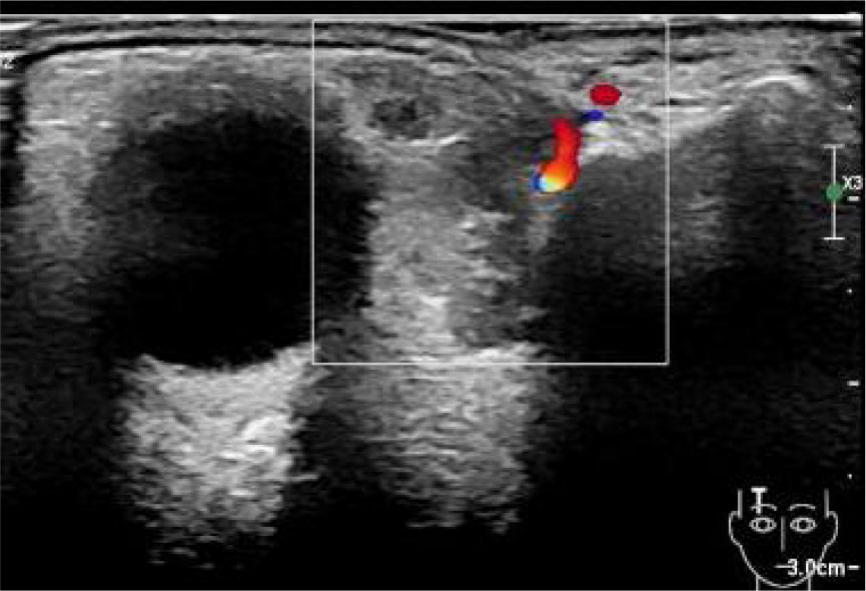
7 days	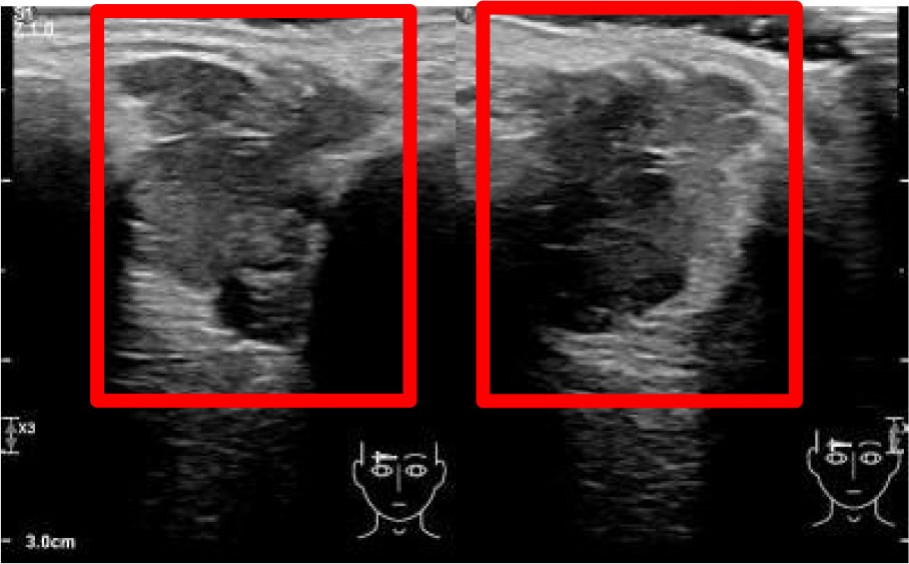	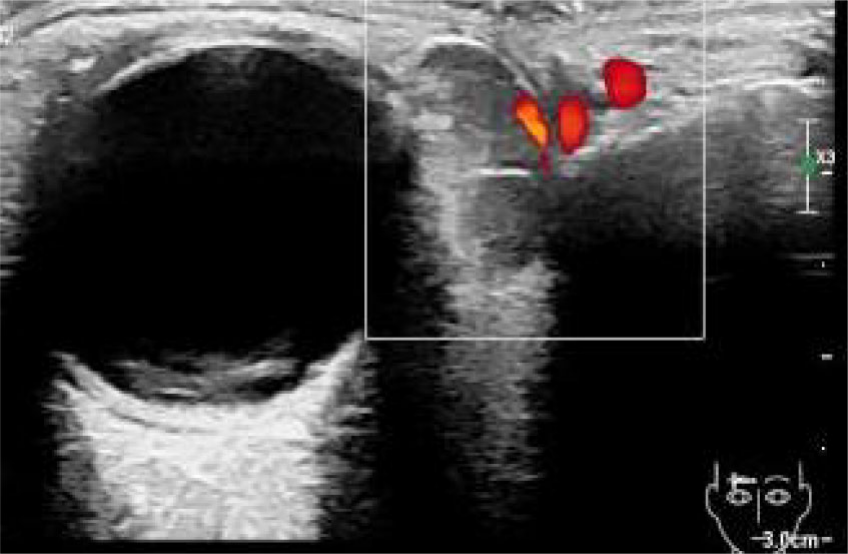
12 days	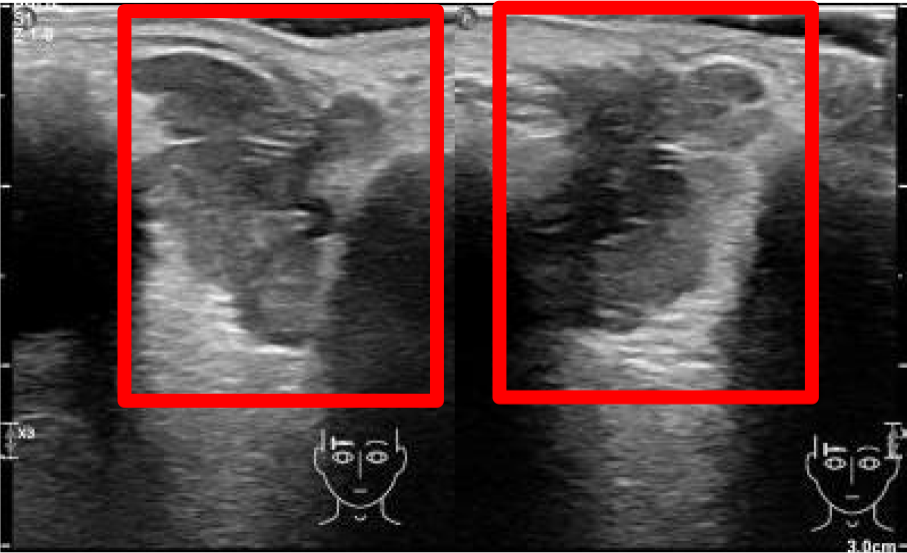	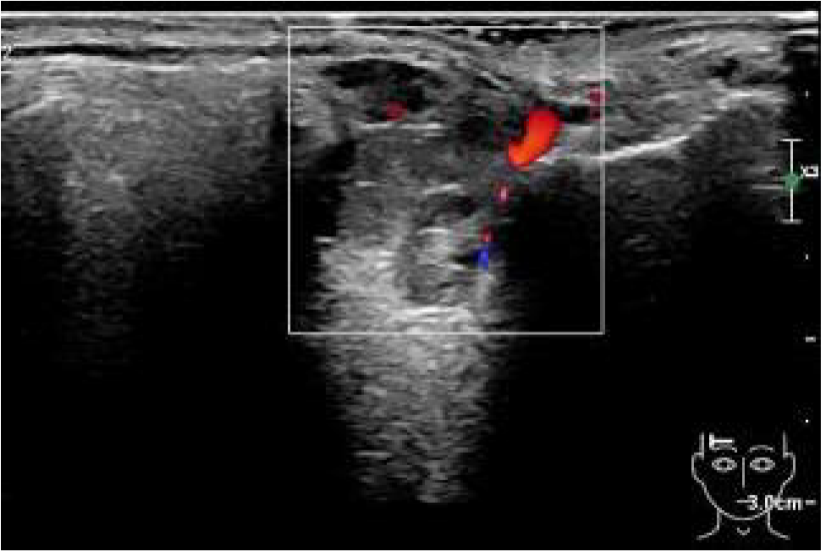
14 days	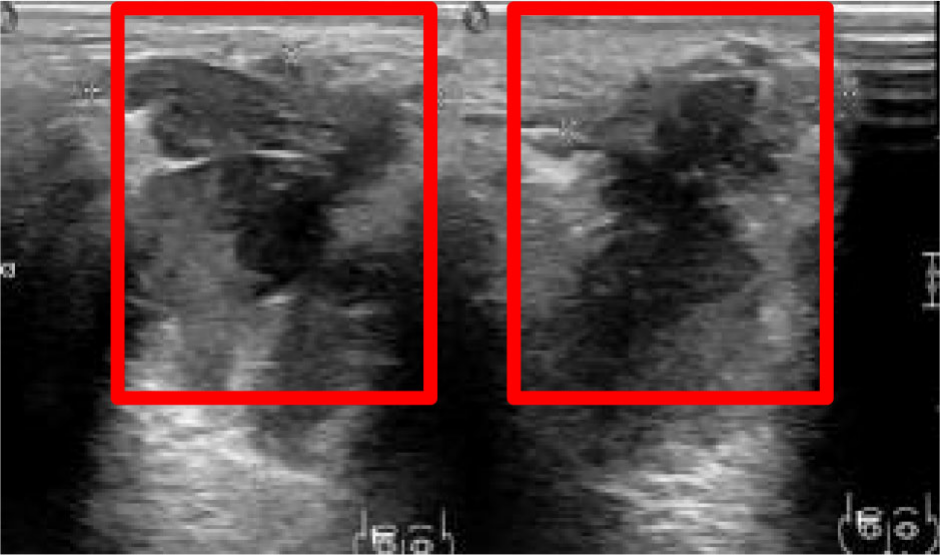	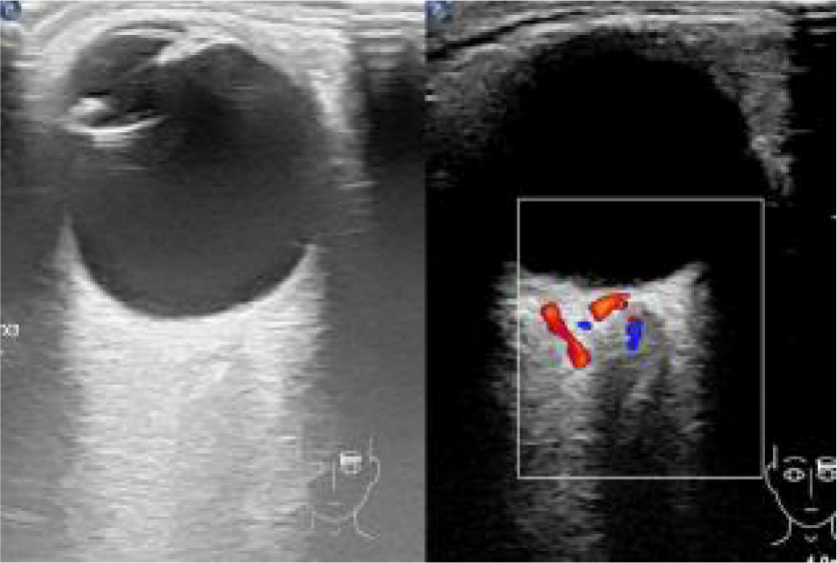

Although color Doppler ultrasound measurements are performed using the same device by the same operator, measurement errors inherent to the equipment must be considered. Therefore, alongside reviewing clinical data, a medical history was taken regarding the patient’s clinical symptoms and signs, as detailed in **Table 5**.

**Table 5:** Clinical symptoms and signs of patients.

Time	Symptoms and signs
Prior to the bloodletting	The patient reports mild swelling in the right eye, accompanied by a slight pressure sensation in the eyeball and a foreign body sensation above the eye. When bending the head downward, the VM enlarges due to gravitational force. Routine ophthalmic examination reveals restricted upward eye movement, with mild ecchymosis visible on the skin overlying the VM.
During the bloodletting procedure	The patient noted a sudden reduction in the size of the VM adjacent to the eyeball, with no further pressure on the eyeball, and the eye appeared normal. The bruising around the eyes gradually faded as the amount of blood drawn increased.
Immediately after the bloodletting is completed	Ophthalmologic examination showed no obstruction during upward movement of the eyeball, and the surface skin at the VM site was slightly bruised but concealed, with no other visible signs. The VM is barely visible.
Within one week after the bloodletting	The patient reports relatively flexible eye movements without gravitational resistance, no sensation of foreign bodies in the orbit, and no difference from the normal left eye. The external appearance of the right eye socket shows no abnormalities.
One week after bloodletting	The patient’s right eye socket began developing a small bruise, which gradually expanded over time. A sensation of pressure from a heavy object around the eye socket emerged, intensifying with prolonged duration. When bending the head downward, the VM exhibited enlargement. Typically, it takes approximately one week to return to the pre-treatment condition.

### Ethical and safety considerations

Written informed consent was obtained from the patient prior to study procedures and manuscript preparation. All procedures were performed under the supervision of a TCM ophthalmologist to minimize vascular injury. Patients were monitored for 20 minutes post-procedure to ensure the absence of bleeding or hematoma formation. Minor ecchymosis around the puncture site was considered a normal response. Strict aseptic techniques were maintained before and after puncture to prevent unnecessary infection. No adverse events occurred during the study.

## Discussion and conclusions

The patient was a young woman. Previous studies suggest that orbital VMs in females are prone to progressive growth with age, occasionally undergoing a phase of rapid expansion (18). Therefore, controlling the growth of periorbital VM was prioritized in this patient. Ocular ultrasound has been shown to be effective for monitoring VMs and associated vascular dynamics (45). To reduce patient exposure to ionizing radiation, color Doppler ultrasound was employed to monitor the orbital VM. Visual acuity remained unaffected, and intraocular pressure and refractive measurements were within normal limits.

Following six months of Taiyang acupoint (EX-HN5) bloodletting therapy, the VM demonstrated no significant enlargement, suggesting a potential effect of this intervention on lesion size. Pre-treatment, the VM measured 1.70×1.67×1.27 cm and exhibited a rich vascular supply. Immediately following bloodletting, the VM decreased to 1.60×1.57×1.25 cm, accompanied by a marked reduction in blood flow. One hour post-bloodletting, the VM measured 1.50×1.48×1.19 cm, demonstrating a continued decrease in size, as illustrated in the line graph. Two hours after treatment, the VM measured 1.46×1.48×1.18 cm, with a deceleration in the rate of reduction compared to earlier time points. Five hours post-procedure, the VM measured 1.58×1.57×1.21 cm, indicating a slight increase, though not returning to baseline dimensions. This transient increase may have been influenced by factors such as head position, ingestion of food, or other conditions affecting blood flow. Between five hours and seven days post-bloodletting, the VM gradually decreased in size, returning to the two-hour measurement, as illustrated in the line graph-a phenomenon not anticipated by the investigators. Color Doppler analysis indicated a gradual increase in blood flow after 24 hours, suggesting partial restoration of vascular perfusion. MRI angiography was not performed to evaluate specific feeding vessels; changes in blood flow were assessed primarily via color Doppler ultrasound. On day 7, the VM measured 1.44×1.44×1.22 cm; on day 12, 1.60×1.50×1.37 cm; and by day 14, 1.61 ×1.55×1.30 cm. The VM showed a tendency to return to its pre-bloodletting size. The patient’s clinical symptoms and signs indicated that prior to the auricular meridian bloodletting therapy, the patient reported mild swelling of the right eye, slight pressure on the eyeball, and a foreign body sensation above the eye. When looking downward, the VM would expand and enlarge due to gravitational force. Routine ophthalmic examination revealed restricted upward eye movement and mild bruising on the skin overlying the VM. During the bloodletting process, the patient observed the vascular tumor adjacent to the eyeball suddenly shrink, eliminating ocular pressure and restoring normal appearance. The ocular bruising gradually faded as bloodletting progressed. Within one week post-treatment, the patient reported unrestricted ocular mobility without gravitational obstruction, no foreign body sensation in the orbit, and no difference from the normal left eye. No abnormalities were observed on the surface of the patient’s right eye socket. One week after bloodletting, a small area of bruising appeared on the surface of the right eye socket. Over time, the bruising expanded, and a sensation of pressure from a heavy object developed around the socket, with the gravitational sensation intensifying. When the patient lowered their head, enlargement of the VM was visible. It typically took one week for the condition to return to its pre-treatment state.

These observations indicate that Taiyang acupoint (EX-HN5) bloodletting therapy produces immediate and significant physiological effects on orbital VM. The effects were not solely subjective, but corroborated by objective color Doppler ultrasound measurements and patient-reported symptoms. Tumor volume continued to decrease within two hours post-treatment, concomitant with reduced blood flow signals observed via color Doppler ultrasound. These findings strongly suggest that bloodletting therapy directly modulates tumor blood volume. Patient-reported alleviation of ocular pressure, restoration of ocular mobility, and resolution of ecchymosis were consistent with objective measurements, indicating that reduced intralesional blood volume directly decreases pressure exerted by the tumor on surrounding tissues. Bloodletting therapy appears particularly effective for small, superficial periorbital VMs by promoting vascular recovery and reducing intralesional pressure, thereby improving patient quality of life. Clinical assessment confirmed that the VM did not affect adjacent nerves or orbital bones, supporting conservative management. The therapy effectively alleviated symptoms induced by the mass effect of the VM within a short-term observational window. These results provide preliminary evidence supporting its use as a palliative, symptom-targeted intervention. The intervention may also complement conventional therapies prior to interventional or surgical procedures, reducing lesion volume and potentially decreasing procedural complexity. For patients unable to tolerate conventional therapies and requiring rapid symptom relief, Taiyang acupoint bloodletting may be considered as an adjunctive option under informed consent. This case demonstrates the dynamic course from onset of effect, transient fluctuation, to recovery, supporting the feasibility of evaluating the efficacy of such interventions. It provides a detailed, contemporary clinical account of this TCM approach, serving as a reference for its potential application. However, for larger or deeper VMs, the efficacy of bloodletting therapy may be limited, with optimal effects often achieved in combination with other treatment modalities. For VMs involving nerves or bony structures, interventions should only be performed under clinician supervision with professional approval.

### Current standard of care and contextualization

The current standard of care for periorbital venous malformations (VM) and hemangiomas is well established, with core treatment modalities including sclerotherapy, surgical excision, pulsed dye laser therapy, and targeted pharmacological interventions (11–16). These conventional approaches aim to control or eradicate lesions but are associated with inherent limitations, including invasive trauma, risk of orbital structural injury, postoperative scarring, and recurrence in complex cases. It is important to clarify that Taiyang acupoint (EX-HN5) bloodletting, as described in this study, serves as an adjunctive, symptom-targeted intervention rather than a definitive curative therapy. This intervention primarily targets relief of clinical symptoms—including periorbital congestion, swelling, cyanosis, and associated discomfort—by improving local venous drainage, without attempting radical ablation of the vascular lesions. Providing this contextualization strengthens the clinical relevance of the study and ensures a balanced perspective on the role of Taiyang acupoint bloodletting in the comprehensive management of periorbital VMs and hemangiomas. To further enhance the therapeutic value of bloodletting therapy for periorbital VM, future studies should investigate underlying mechanisms, optimize patient selection and individualized treatment, and conduct multicenter clinical trials. The precise therapeutic mechanisms remain unclear. Future studies should examine its effects on VM tissue and local blood circulation, particularly regarding vascular endothelium, smooth muscle, and microcirculation, to provide stronger theoretical support for clinical application. Considering variability in clinical presentation and disease duration, further research is needed to identify VM patient subgroups most suitable for bloodletting therapy and to develop tailored treatment protocols. Optimizing patient selection may enhance therapeutic efficacy and minimize adverse effects. Existing studies on bloodletting therapy are mainly small-scale clinical observations and lack large-scale, multicenter, randomized controlled trials. Future research should conduct high-quality, large-sample studies to reliably evaluate efficacy and establish a robust scientific basis for broader clinical application.

### Key limitations

According to the CARE guidelines, MRI was not performed in this case, limiting detailed documentation of diagnostic methods, diagnostic reasoning (including differential diagnosis), and prognostic characteristics, thereby constraining the study’s results. Furthermore, the conclusions of this study are applicable only to adult patients with similar conditions and may not be generalizable. Therapeutically, as a conservative intervention, bloodletting demonstrates limited efficacy and may be considered primarily as an observational treatment. This approach is not recommended for patients with comorbidities or elderly individuals due to potential risks, including bleeding and increased vascular fragility.

## Conclusion

This case demonstrates that Taiyang acupoint (EX-HN5) bloodletting therapy produces significant clinical effects in periorbital VMs. As a TCM technique, it shows potential particularly for early-stage, less symptomatic patients, providing preliminary clinical evidence supporting its application in the management of periorbital VMs.

## Data Availability

The raw data supporting the conclusions of this article will be made available by the authors, without undue reservation.
